# A Study of Factors Contributing to the Nutritional Status of Elderly People Receiving Home Care

**DOI:** 10.3390/nu16183135

**Published:** 2024-09-17

**Authors:** Eirini Stratidaki, Enkeleint A. Mechili, Christina Ouzouni, Athina E. Patelarou, Konstantinos Giakoumidakis, Aggelos Laliotis, Evridiki Patelarou

**Affiliations:** 1 Department of Nursing, School of Health Sciences, Hellenic Mediterranean University, 71004 Heraklion, Crete, Greece; apatelarou@hmu.gr (A.E.P.); kongiakoumidakis@hmu.gr (K.G.); epatelarou@hmu.gr (E.P.); 2 Department of Healthcare, Faculty of Health, University of Vlora, 9401 Vlora, Albania; mechili@univlora.edu.al; 3 School of Medicine, University of Crete, 71500 Heraklion, Crete, Greece; 4 Occupational Therapy Department, University of West Attica, 12243 Egaleo, Greece; ouzouni@uniwa.gr; 5 Department of General Surgery, Venizeleio General Hospital, 71409 Heraklion, Crete, Greece; laliotisac@gmail.com

**Keywords:** nutrition, malnutrition, nutrition status, elderly people

## Abstract

(1) Background: Nutrition is a critical aspect of health and well-being in the elderly population, as physiological changes associated with aging can impact nutrient utilization and dietary needs. The aim of this study was the assessment of nutritional screening and associated factors among community-dwelling elderly people. (2) Methods: This study is the first phase of an intervention trial of people aged 65 years and over who received primary health services and resided in the municipality of Archanes Asterousia in Crete, Greece. Nutritional risk was assessed using the Mini Nutritional Assessment. Diet-related factors were analyzed, including health status (oral hygiene, depression, cognitive decline, impaired functioning, quality of life), social factors (educational attainment, marital status, type of work before 60 years), and lifestyle factors (smoking, drinking, diet). (3) Results: A total of 730 elderly people were evaluated (males, 31.5%), with an average age (±SD) of 76.83 (±6.68) years. MNA was found to have a statistically significant connection with assessment of oral hygiene, mental state, Charlson comorbidity, functional independence (assessed with the Barthel scale), and quality of life. The exception was geriatric depression (GDS), with which no statistically significant association was found (*p* > 0.05). Nutritional risk analysis revealed 379 participants (51.9%) to be adequately nourished, 205 (28.1%) to be at risk of malnutrition, and 146 (20.0%) to be malnourished. (4) Conclusions: These results clearly demonstrated the key factors that contribute to the nutritional screening of elderly people and need to be addressed by health authorities and social services.

## 1. Introduction

The number of elderly people is expected to increase from 900 million to 2 billion between 2015 and 2050 (from 12% to 22% of the world’s total population), with developing countries accounting for most of this increase. As a result, the elderly are the fastest growing population group [1,2,3]. The health or lack thereof in this rapidly growing population not only affects the individuals themselves but also has serious implications for demands on health care and other social resources. Several studies examining lifestyle and health variables have suggested that food consumption is an important predictor of life expectancy [4]. In older people, poor nutrition can increase the incidence and severity of disease, accelerating the loss of independence. Therefore, it is important to understand the factors that influence the food choices of older adults to help ensure healthier diets [5].

Elderly people receiving home care are at a significantly higher risk of malnutrition [6]. Health professionals, particularly dietitians, play a vital role in addressing nutritional challenges [7]. Nutritional interventions, including screening and assessment, have been proposed to improve health outcomes and reduce healthcare costs among malnourished elderly people [8]. Effective pre-screening tools such as the Mini Nutritional Assessment and interventions, including personalized nutritional advice and home-delivered meals, can play an important role in addressing this concern [9]. While integrated home care provides broader health benefits, the specific effects on nutritional status have not been detailed, highlighting the need for targeted interventions to mitigate the risk of malnutrition. Additionally, the involvement of family caregivers in these interventions may play a critical role in improving the overall nutritional status of elderly home care recipients [10].

The use of weight loss is an inadequate measure of nutritional intake. As weight loss can be caused by diseases that increase metabolism, this measure may not be sufficiently specific or necessary to reduce energy and nutrient consumption. Furthermore, when energy intake is adequate but nutrition is deficient in certain nutrients, weight loss may not occur [11].

Malnutrition in elderly home care recipients is influenced by oral health, swallowing function, cognition, and activities of daily living [12]. Socioeconomic factors also affect nutritional status, with malnutrition being more common in lower socioeconomic groups [13,14]. One study did not find a direct association between oral health and malnutrition [13], while another study suggested that maintaining oral health and using dentures support ADL and nutrition [12]. Nutritional interventions are recommended to improve health outcomes and reduce health care costs in malnourished older adults [8]. Addressing physical health, cognitive ability, and socioeconomic status can improve nutritional outcomes and overall health [14,15].

Oral health problems such as pain and abscesses cause difficulties in eating and chewing and therefore have a significant impact on the nutritional status of the elderly [16]. Moreover, elderly people who use dentures must be taught proper home care for both the dentures and the tissues on which they rest, as well as the need for ongoing professional care. Caloric requirements are usually reduced in the elderly due to a decrease in the basal metabolic rate caused by reduced muscle mass and lower levels of exercise. Appetite can also be reduced, leading to insufficient caloric intake and often leading to inadequate consumption of calcium, iron, and zinc, especially in women. However, certain groups of the elderly, such as those who are at home and without access to sunlight, may have insufficient vitamin D and develop an increased risk of falls [17].

Consuming a healthy diet throughout the life-course helps to prevent malnutrition in all its forms as well as a range of noncommunicable diseases (NCDs) and conditions [18]. Investigating the prevalence and determinants of malnutrition in vulnerable groups is critical for proposing strategies to address this problem and provide holistic assistance to older people. Few studies have examined the nutritional status of the elderly population in Greece, and almost no research has been conducted in the study area to date. Studies of community-dwelling older adults aged 65 years or above have shown that the factors influencing nutritional risk differ, indicating the need for more research into the factors that contribute to undernourishment and optimal nutritional status. 

Therefore, we hypothesized that socioeconomic factors such as low education level, living alone, being single, low income level, gender, residence, employment status, lifestyle, degree of independence, cognitive impairment, and depression may be related to the nutritional risk of the elderly. The general objective of the study included the assessment of the nutritional status and associated factors among community-dwelling older adults. The specific goals assessed were:

- To determine the prevalence of poor nutritional status among elderly individuals.
- To assess factors related to the nutritional status of the elderly.

## 2. Materials and Methods

This study is the first phase of an intervention trial. In the current study, we are presenting the data collected between August 2021 and July 2023 in the Municipality of Archanes Asterousia, Prefecture of Heraklion in Crete. Elderly people aged 65 and over enrolled in the “Help at Home” program and the Open Protection Center for the Elderly were approached.

The program “Help at Home” is a social program that provides free care services and aims to improve the quality of life of the people served and the persons responsible for their care. It caters to elderly people aged 65 and over and to people with disabilities who need home help, live in the municipality, and meet the inclusion criteria. The Open Protection Center for the Elderly offer psycho-emotional support, social care, medical, and hospital care to the elderly. Physiotherapy is also offered to those who need it. In addition, occupational therapy and organized entertainment are provided.

As of the 2021 census, the total population of the study area was 18,746, and that of people ≥ 60 years of age was 5484. A convenience sample of all residents over the age of 65 who were registered to the home help program and were eligible and willing to participate were included in the study. In consultation with the coordinating nurse of the center, appointments were scheduled with the elderly at the center at the time and day they were available, upon which the necessary data were recorded and measurements were taken. In addition, home visits were also made to those who were registered in the “Help at Home” program and could not come to the center. During these visits, the measurements of weight, height, and arm and leg circumference were made, and the study questionnaires were completed. Somatometric measurements were taken by the researcher, and the questionnaires were self-completed by the participants, with the assistance of the researcher when needed. The mean duration for gathering all the information needed for the purposes of the study was 25–35 min. In total, 1075 participants were approached, and 730 finally agreed to participate in the study, providing their written consent. The study excluded elderly people with severe dementia, overwhelmed patients, and those who refused to participate in the study. We performed post hoc power analysis with MNA and MMSE, using the same software as the analysis. The power was found to be 99%, while a previous study reported 0.428 [19].

### 2.1. Data Collection

Data were collected using evaluated questionnaires. First, for each of the participants, we collected demographic characteristics such as age, gender, and region of residence. For each of the participants, we measured height to the nearest 0.1 cm using a stadiometer (Gima, Gessate, Italy), arm and circumference leg with a tape measure, and weight using a Seca Scale (Seca, Hanover, MD, USA). Participants were weighed without shoes while wearing light clothing, and the scale was placed on a hard, flat surface. Τhe measurements were all done in the morning.

For the nutritional screening of the elderly, using the Mini Nutritional Assessment Tool (MNA), leg circumference and upper arm circumference were measured by a tape measure. BMI was calculated by dividing body weight in kilograms by height squared. According to the MNA, BMI was scored as 0 for BMI less than 19, 1 for BMI 19 to less than 21, 2 for BMI 21 to less than 23, and 3 for BMI 23 or greater.

In addition, the following rating scales were used:

For the nutritional screening of the elderly, the Greek version of the Mini Nutritional Assessment (MNA) questionnaire was used. This includes four subcategories of elements (anthropometric data, nutritional screening, subjective assessment, and overall assessment of the person). It calculates the person’s nutrition on a scale wherein >24 is considered well-nourished, 17–23.5 is at risk of malnutrition, and an index <17 is considered undernourished [20].

The Oral Health Assessment Tool (OHAT) was used to assess oral hygiene. The tool consists of eight categories (‘lips’, ‘tongue’, ‘gums and tissues’, ‘saliva’, ‘natural teeth’, ‘dentures’, ‘oral cleanliness’, and ‘dental pain’), with three possible scores (0: healthy, 1: some changes present, and 2: unhealthy condition) [21]. 

Disability or degree of functionality or independence were assessed in terms of performing daily activities with the Index for Activities of Daily Living (Barthel). More specifically, the index examines the ability to walk, move, climb stairs, eat, use the toilet, bathe, and perform other personal hygiene, as well as the ease of bowel movements, control of the sphincter, and getting dressed. The total score ranges from 0–100; the lower the values, the greater the disability or reduced functioning or loss of independence [22].

The impairment of cognitive function (cognitive decline), associated with dementia, was assessed with the widely used Mini Mental State Examination scale (MMSE). The total score falls in the range of 0–30, and values > 24 are considered normal. During the exam’s implementation, the participant is asked to perform 5 categories of visual–spatial exercises (cube copying), tasks that assess memory, orientation, and problem solving [23].

For the assessment of geriatric depression, the Greek version of the Geriatric Depression Scale (GDS) was used [24]. The GDS is a standardized scale, consisting of 15 YES–NO questions. A score of 0–5 corresponds to the absence of depressive symptoms, 6–10 to mild depression, and 11–15 to severe depression.

The Charlson Comorbidity Index (CCI) was used to evaluate the degree of incidence of comorbidities [25]. It consists of 15 pathological conditions such as diabetes, hemiplegia, AIDS, myocardial infarction, etc. The degree of severity of multimorbidity is expressed in average values through an online algorithmic formula.

The degree of homebound status, and consequent imperative need for “home care”, was defined based on the elderly person’s ability to leave or move away from their home during the last month, according to the recommendations of a recent multi-population study [26].

For the quality of life of the elderly, the Greek version of the WHOQOL-BREF scale was used, which includes 26 questions concerning 4 dimensions of health (physical, psychological, social relationships, environment) and 4 additional questions concerning nutrition, satisfaction with work, home life, and social life [27].

### 2.2. Statistical Analysis

After the sample was collected, the survey data were entered and analyzed in the statistical program SPSS 25.0 (IBM Statistical Package for Social Sciences for Windows, Version 25.0. Armonk, NY, USA: IBM Corp). The level of statistical significance was set at 5%.

The qualitative variables of the research were described using absolute frequency (n) as well as relative frequency (%). The values used in quantitative variables that met the assumption of normality were the mean (M) and standard deviation (SD), while in those that did not meet the assumption of normality, the median and interquartile range were used. Normality of the variables was tested using kurtosis and skewness.

For correlation analysis, the Pearson r index was used when the assumption of normality was met, while in other cases the Spearman correlation index was used. The χ^2^ test was used to test independence. The *t*-test and Mann–Whitney U test for independent samples were used to investigate differences in means and medians, depending on whether the assumption of normality was met. To investigate the differences when there were more than two categories (e.g., in the case of the vulnerability analyses), the analysis of variance (ANOVA) with post hoc Games–Howell test was used, while in case the assumption of normality was not met, the non-parametric Kruskal–Wallis H test was used. For the logistic regression model, and more specifically the input of the variables, the method applied was “Enter”.

### 2.3. Ethical Approval and Consent to Participate

The study was conducted in accordance with the Declaration of Helsinki and approved by the Research Ethics Committee of the Hellenic Mediterranean University. All rules on the protection of individuals regarding the processing of personal data were followed in compliance with the requirements of the General Data Protection Regulation (GDPR) and the Data Protection Act of 2019.

## 3. Results 

### 3.1. Basic Characteristics of Participants

A total of 730 elderly people aged 65–96 years (mean = 76.83, standard deviation (SD) = 6.68) who met the inclusion and exclusion criteria were recruited in this study (Table 1). Of the sample, 68.5% consisted of women and came from Greece (97.9%). Furthermore, 70.1% of participants stated that their individual annual income exceeds €4500, while 60.6% stated that their individual annual income exceeds €9475. As regards the level of education, 69.2% had a low level of education without knowledge of another foreign language (83.8%). In the majority of elderly people, their work before retirement was manual (79.6%), and their marital status was married for 57.3% and widowed/divorced for 40.0%. When conducting the survey, 40.8% said they lived alone, while 59.2% said they lived with another person in the same place. The majority had experienced a decline in the last month (92.1%), and 9.7% were in home confinement, 28.9% in semi-confinement, and 61.4% were not in confinement.

Descriptive measures of all variables used in the research are next presented. Regarding Nutritional Risk Assessment (MNA), the mean was M = 22.32 and the standard deviation SD = 5.83. For the Oral Hygiene Assessment (OHAT), the mean was M = 11.28, while the standard deviation was SD = 2.85. The Mental Status Examination (MMSE) was found to have M = 27.78 and SD = 1.69. The Charlson Comorbidity Index (CCI) showed M = 4.96 and SD = 1.72, while in percentage values, M = 31.57 and SD = 32.74. The Barthel scale showed M = 18.73 and SD = 2.09, while in values from 0–100, M = 93, 65 and SD = 10.43. The Geriatric Depression Scale rendered M = 8.12 and SD = 3.90. Regarding the Quality of Life Questionnaire (WHOQOL-BREF), total quality of life had a mean M = 12.55 and standard deviation SD = 3.30, physical health had M = 12.43 and SD = 2.15, psychological health had M = 12.85 and SD = 2.29, social relations had M = 12.70 and SD = 3.53, and environment had M = 12.85 and SD = 3.13 (Table 2).

Correlation analysis was conducted using the Pearson r index and the Spearman rho index (for variables where the normality hypothesis was not met). 

As presented in Table 3, statistically significant associations (positive or negative) were found in all variables studied with weak, moderate, or strong relevance, except for geriatric depression (GDS), for which no statistically significant association was found (*p* = 0.380). Nutritional Risk (MNA) correlates positively with Oral Hygiene Assessment (OHAT) with moderate relevance (r = 0.450, *p* < 0.001), and with the Mental Status Examination (MMSE) with weak relevance (r = 0.259, *p* < 0.001). A positive correlation of strong relevance was also found between Nutritional Status (MNA) and the Barthel functional independence scale (r = 0.822, *p* < 0.001). 

In contrast, a negative weak relevance association was found between Nutritional Status (MNA) and Charlson Comorbidity Index (CCI) (r = −0.377, *p* < 0.001). Finally, all scales of the WHOQOL-BREF were positively correlated with Nutritional Status (MNA) with moderate or strong relevance (*p* < 0.001 for all associations). 

### 3.2. Nutritional Risk of Elderly People

In terms of the Nutritional Risk Assessment (MNA), 379 participants (51.9%) were found to be normal, 205 (28.1%) at risk of malnutrition, and 146 (20.0%) malnourished.

### 3.3. Factors Associated with Malnutrition among the Elderly

Nutritional risk analysis revealed 379 participants (51.9%) to be adequately nourished, 205 (28.1%) to be at risk of malnutrition, and 146 (20.0%) to be malnourished. Malnourished patients were older, were more often widowed/divorced or unmarried, lived alone, had had falls in the last month, and had a homebound status of confined (Table 1).

A one-unit increase in the Barthel variable is associated with a decrease in the relative log odds of being at risk of malnutrition vs. normal. In the remaining variables, no statistically significant differences were found. That is, there was no differentiation of geriatric depression, oral hygiene, mental status, comorbidity, functional independence, and quality of life depending on the nutritional status of the elderly (Table 4). Pearson goodness-of-fitness showed *p* < 0.001, while deviance was found with *p* > 0.999. Nagelkerke R^2^ was found as 0.737. AIC = 784.64, BIC 885.689, *p* < 0.001. Generally, the model fits well.

## 4. Discussion

This study aimed to assess the nutritional risk and associated factors among community-dwelling elderly people in the wider area of Heraklion, Crete, Greece. According to the results of the current work, we found that 51.9% of the elderly were of normal nutrition, 28.1% at risk of malnutrition, and 20.0% categorized as malnourished. Additionally, the MNA mean was found at a level of M = 22.32 and the standard deviation SD = 5.83. MNA correlation was found to be statistically significant with the scales of assessment of oral hygiene, geriatric depression, mental state, Charlson comorbidity, functional independence (assessed with the Barthel scale), and quality of life. Malnourished participants were older, were more often widowed/divorced or unmarried, lived alone, had had falls in the last month, and had a homebound status of confined. These results clearly demonstrate the key factors that contribute to the nutritional status of older people and need to be addressed by health authorities and social services to help them. 

In contrast to some studies, we did not find a correlation between nutritional risk and gender, economic status, or education level. In these studies, the female gender, being associated with lower education and income, has usually been an indicator of malnourishment [28,29,30,31]. However, other studies confirm our results [32]. Higher age is a clear indicator and strongly correlated with a worse nutritional risk [33,34]. Another indicator of being malnourished is the family/marital status [28,31]. People that live alone are at a higher risk of being malnourished. Living alone increases the levels of depression and social isolation and deteriorates the quality of life. Moreover, preparation of food in these cases becomes less interesting. Provision of free meals for people living alone could improve the nutrition level. 

The mean of the MNA differs with a study conducted in Greece recently [28]. This study shows a lower MNA (MNA score = 9.5; SD = 3.9), which means a better nutritional status among people over 65 years old than in the current work. However, it is important to mention that the studies were conducted in different places of residence in Greece (the current one on the island of Crete and the other on the mainland). A study in Italy among older people showed that 65% of them were at risk of malnutrition and 10% were malnourished [35]. Another study found that 62.4% of elderly participants were at risk of malnutrition and 9.3% were of normal nutritional status [36]. A study conducted in China reported that among elderly people living in nursing homes, 46.4% had a normal nutritional status while 39.2% and 14.4% were at risk of malnutrition and malnourished, respectively [32]. However, another Chinese study classifies the elderly as 2.2% malnourished, 38.4% at risk of malnutrition, and 59.4% well-nourished [33]. A study that took place in Guadeloupe (French West Indies) among elderly people living in foster families reported that 28% of them were malnourished [37]. A study in Switzerland showed that among community-dwelling, dependent older adults, 60.0% were classified as normally nourished, 10.8% as malnourished, and 30.4% at risk of malnutrition [38]. Another work among a geriatric population found 38.4% of the population with a normal status of nutrition, 47.8% at risk of malnutrition, and 14% with a malnourished status [39]. The differences in the studies could be due to the different methodological approaches (population age, setting, population health status, scope of the study, time and method of data collection), socioeconomic status of the sample and among countries, as well as the age of the participants. Several studies included people older than 70 or 75, while others focused on a younger population (like the current one). Despite the differences between the studies, it is evident that around half of elderly people are at high risk of malnutrition or are malnourished. It is very important for the diet of the elderly to be rich in fruits, vegetables, and proteins. Moreover, a better diet can help in emotional balance, as has been reported by a study conducted in Crete, Greece [40]. 

Oral health status is a key indicator of the nutritional risk of the elderly. Better oral health significantly ameliorates nutritional risk. One study shows that poor oral health significantly increases the odds of malnutrition (around six times higher) [29]. Usually, elders do not focus much on their oral health, and many of them have a low number of teeth or use prostheses that impact their chewing efficiency [40]. In general, elderly people with a higher number of teeth have better nutrition in comparison to those with fewer teeth [41]. This can contribute to the types of food that they can consume, potentially excluding them from many that could be useful for their health and well-being. Xerostomia in the elderly is associated with the risk of malnutrition [42]. Taking care of oral health and providing regular dental care can help significantly in the nutritional risk of the elderly. Moreover, it is recommended that elderly people receive free-of-charge dental services. Social services should conduct regular discussions with them about the importance of oral health. Replacement of lost teeth can also improve the MNA score [43]. 

Several studies in the literature confirm the existence of a correlation between nutritional status and mental health [31,37,44,45]. Elderly people usually suffer from depression and mental health problems. Living alone or living in geriatric centers, having low or no contribution to society, social isolation, having chronic conditions, being visited rarely by family members, and financial problems are some of the key factors that contribute to mental health status [46]. It is clear that regular screening for anxiety and depression is necessary for this vulnerable population. When the results are positive for these, referral to the appropriate services and antidepressive treatment should be offered. Training of healthcare and non-healthcare personnel for recognition and diagnosis of early anxiety and depression symptoms is crucial.

Similar to this study, the literature shows that people living with chronic conditions have worse nutritional status [31,32,47]. The higher the number of chronic diseases, the higher the possibility of being malnourished. Living with chronic conditions leads to a need for more drugs, and this can have an impact on gastrointestinal function. Deterioration of the gastrointestinal system and presence of ulcer make elderly people reluctant to consume food, which leads them to malnutrition. A literature review has focused on this issue, noting that implementation of preventive measures can help [48]. 

Functionality degree and independence to do things alone was seen in the current study to have an impact on nutritional status. Elderly people with low levels of independence and functionality suffer more from malnutrition. This echoes studies conducted in Italy and Peru which show a correlation between MNA and the Barthel scale [47,49]. Keeping them active and in good physical shape is important for this population. BMI is another simple indicator that can help in the assessment in this case. 

Better quality of life is strongly connected with better nutritional status. A very recent study in Greece has found similar results, with a positive relation between all quality-of-life domains and nutritional status [28]. Similarly, a study in Vietnam among elderly people living in rural areas reported a correlation between all quality-of-life domains and nutritional status [30]. The use of food intake as an indicator of quality of life is important for prevention and improvement. However, this is a two-sided direction as good quality of life lowers nutritional risk and vice versa. Moreover, the lower the nutritional risk, the better the functionality and independence. 

All the indicators above are correlated with each other, and holistic approaches are needed to address the nutritional risk of elderly people. It is more than evident that improvement in quality of life can lead also to lower levels of depression and greater functionality. Additionally, improvement of oral health can impact nutritional status as well as quality of life. Due to the aforementioned, it is of paramount significance that these indicators not be analyzed and addressed individually but in a synergistic and comprehensive way. 

### Strengths and Limitations

To our best knowledge, this is the first study conducted in Greece that assesses the nutritional status of elderly people by taking into consideration so many different scales (six in total). This study clearly demonstrates the key factors that contribute to the malnutrition of the elderly, and based on this, intervention can be prepared at an individual, family, and community level. Additionally, the large sample size and high participation rates are very strong points of the current article. 

As all studies do, this one has some limitations. Its cross-sectional nature and collecting data only from one region of Greece limit our ability to generalize the current results. Moreover, most of the participants were of female gender, and this is also a limitation for external validity. Additionally, the low education level of the participants could have impacted their understanding of the questions. Future studies should consider more settings in Greece. Another critical point for future studies is the follow-up of the participants after implementing some interventions.

## 5. Conclusions

This study found that 51.9% of the elderly were of normal nutrition, while 28.1% were categorized as at risk of malnutrition and 20.0% as malnourished. MNA was found to have a statistically significant correlation with assessment of oral hygiene, geriatric depression, mental state, Charlson comorbidity, functional independence (assessed with the Barthel scale), and quality of life. Key factors that contributed to nutritional status were age, family, and homebound status. Gender, socioeconomic status, smoking status, and education level did not show statistically significant impact on the nutritional level. These results clearly demonstrate the key factors that contribute to the nutritional risk of the elderly and need to be addressed by health authorities and social services. Better collaboration and regular training of medical and non-medical professionals, improvement of social services, and systematic provision of dental and mental health services in community dwellings are key measures that can reduce the nutritional risk of the elderly.

## Figures and Tables

**Table 1:** Demographics and other characteristics stratified according to nutritional risk.

		Normal (12–14 Points) (*n* = 379)	At Risk of Malnutrition (8–11 Points) (*n* = 205)	Malnourished (0–7 Points) (*n* = 146)	*p*-Value
Age (years, mean ± SD)		74.59 ± 6.30	78.11 ± 6.05	80.88 ± 6.09	<0.001 ^‡^
Gender (*n*, %)					0.438
	Male (230, 31.5%)	127 (33.5%)	62 (30.2%)	41 (28.1%)	
	Female (500, 68.5%)	252 (66.5%)	143 (69.8%)	105 (71.9%)	
Origin (*n*, %)					0.290 ^†^
	Greece (715, 97.9%)	370 (97.6%)	201 (98.0%)	144 (98.6%)	
	Other (15, 2.1%)	9 (2.4%)	4 (2.0%)	2 (1.4%)	
Annual Individual Income (€) (*n*, %)					0.736
	>4500 (512, 70.1%)	270 (71.2%)	143 (69.8%)	99 (67.8%)	
	<4500 (218, 29.9%)	109 (28.8%)	62 (30.2%)	47 (32.2%)	
Annual Family Income (€) (*n*, %)					0.858
	>9475 (432, 60.6%)	229 (61.2%)	121 (60.8%)	82 (58.6%)	
	<9475 (281, 39.4%)	145 (38.8%)	78 (39.2%)	58 (41.4%)	
Program (*n*, %)					0.656
	Help at Home (553, 75.8%)	283 (74.7%)	160 (78.0%)	110 (75.3%)	
	Center for Open Protection of the Elderly (KAΠH) (177, 24.2%)	96 (25.3%)	45 (22.0%)	36 (24.7%)	
Smoker (*n*, %)					0.529
	Current (106, 14.5%)	62 (16.4%)	24 (11.7%)	20 (13.7%)	
	Ex (327, 44.8%)	167 (44.1%)	98 (47.8%)	62 (42.5%)	
	Never (297, 40.7%)	150 (39.6%)	83 (40.5%)	64 (43.8%)	
Educational level (*n*, %)					
	Lower (505, 69.2%)	258 (68.1%)	134 (65.4%)	113 (77.4%)	
	Middle (208, 28.5%)	113 (29.8%)	66 (32.2%)	29 (19.9%)	
	Higher (17, 2.3%)	8 (2.1%)	5 (2.4%)	4 (2.7%)	
Knowledge of a Foreign Language (*n*, %)					0.621
	No (612, 83.8%)	315 (83.1%)	177 (86.3%)	120 (82.2%)	
	English (36, 4.9%)	20 (5.3%)	9 (4.4%)	7 (4.8%)	
	German (44, 6.0%)	23 (6.1%)	8 (3.9%)	13 (8.9%)	
	Other (38, 5.2%)	21 (5.5%)	11 (5.4%)	6 (4.1%)	
Job (*n*, %)					0.356
	Mental work (64, 8.8%)	38 (10.0%)	16 (7.8%)	10 (6.8%)	
	Manual work (581, 79.6%)	304 (80.2%)	163 (79.5%)	114 (78.1%)	
	Combination of manual and mental work (85, 11.6%)	37 (9.8%)	26 (12.7%)	22 (15.1%)	
Family Status (*n*, %)					0.001
	Unmarried (20, 2.7%)	7 (1.8%)	6 (2.9%)	7 (4.8%)	
	Married (418, 57.3%)	238 (62.8%)	117 (57.1%)	63 (43.2%)	
	Widowed/divorced (292, 40.0%)	134 (35.4%)	82 (40.0%)	76 (52.1%)	
Do you live alone? (*n*, %)					<0.001
	Yes (298, 40.8%)	132 (34.8%)	85 (41.5%)	81 (55.5%)	
	No (432, 59.2%)	247 (65.2%)	120 (58.5%)	65 (44.5%)	
Have you had falls in the last month? (*n*, %)					<0.001
	No (672, 92.1%)	376 (99.2%)	191 (93.2%)	105 (71.9%)	
	Yes (58, 7.9%)	3 (0.8%)	14 (6.8%)	41 (28.1%)	
Homebound status (*n*, %)					<0.001
	Confined (71, 9.7%)	0 (0.0%)	10 (4.9%)	61 (41.8%)	
	Semi-confined (211, 28.9%)	21 (5.5%)	128 (62.4%)	62 (42.5%)	
	Non-confined (448, 61.4%)	358 (94.5%)	67 (32.7%)	23 (15.8%)	

Notes. Values are refer to absolute and relative frequencies (%) or means ± standard deviations (SD). *p*-value is computed using chi-square test, ^†^ chi-square test with Monte Carlo simulation, or ^‡^ *t*-test.

**Table 2:** Descriptive measures of the variables under study.

	M	SD
Mini Nutritional Assessment (MNA)	22.32	5.83
Oral Health Assessment Tool (OHAT)	11.28	2.85
Mini Mental State Examination (MMSE)	27.78	1.69
Charlson Comorbidity Index (CCI)	4.96	1.72
Charlson Comorbidity Index (CCI) %	31.57	32.74
Barthel scale	18.73	2.09
Barthel scale (0–100)	93.65	10.43
Geriatric Depression Scale (GDS)	8.12	3.90
Quality of life (WHOQOL-BREF)		
Overall quality of life/general health	12.55	3.30
Physical health	12.43	2.15
Psychological health	12.85	2.29
Social relationships	12.70	3.53
Environment	12.85	3.13

**Table 3:** Correlation between Nutritional Risk Assessment (MNA) and all questionnaire scales.

	Nutritional Risk Assessment (MNA)	
	R	*p*
Oral Health Assessment Tool (OHAT)	0.450 ***	<0.001
Geriatric Depression Scale	−0.033	0.380
Mini Mental State Examination (MMSE)	0.259 ***	<0.001
Charlson Comorbidity Index (CCI)	−0.377 ***	<0.001
Charlson Comorbidity Index (CCI) %	0.332 ***	<0.001
Barthel scale	0.822 ***^†^	<0.001
Barthel scale (0–100)	0.822 ***^†^	<0.001
Quality of life (WHOQOL-BREF)		
Overall quality/general health	0.532 ***	<0.001
Physical health	0.626 ***	<0.001
Psychological health	0.615 ***	<0.001
Social relationships	0.687 ***	<0.001
Environment	0.707 ***	<0.001

Notes. The values refer to a correlation index of Pearson or ^†^ Spearman rho. *** *p* < 0.001.

**Table 4:** Multinomial logistic regression with Mini Nutritional Assessment (MNA) as dependent variable.

		Model Fitting Criteria	Likelihood Ratio Tests		OR (95% CI) At Risk of Malnutrition vs. Normal	OR (95% CI) Malnourished vs. Normal
		−2 Log Likelihood of Reduced Model	Chi-Square	*p*-Value		
Oral Health Assessment Tool (OHAT)		741.538	0.896	0.639	0.971 (0.866, 1.089)	0.935 (0.812, 1.078)
Geriatric Depression Scale (GDS)		740.740	0.098	0.952	0.999 (0.931, 1.072)	0.988 (0.899, 1.085)
Mini Mental State Examination (MMSE)		744.049	3.407	0.182	0.877 (0.729, 1.055)	0.989 (0.782, 1.251)
Charlson Cormobidity Index (CCI)		744.391	3.750	0.153	0.923 (0.760, 1.120)	1.102 (0.856, 1.417)
Barthel scale (0–100)		927.085	186.444	<0.001	0.071 (0.040, 0.125)	0.050 (0.028, 0.089)
Quality of life (WHOQOL-BREF)						
	Overall quality of life/general health	740.917	0.275	0.872	0.971 (0.860, 1.095)	0.963 (0.816, 1.135)
	Physical health	740.684	0.042	0.979	0.990 (0.792, 1.237)	0.968 (0.707, 1.325)
	Psychological health	742.669	2.028	0.363	0.933 (0.759, 1.147)	0.821 (0.622, 1.084)
	Social relationships	743.348	2.707	0.258	0.905 (0.764, 1.072)	0.826 (0.657, 1.039)
	Environment	741.955	1.314	0.518	0.963 (0.768, 1.207)	0.852 (0.631, 1.151)

## Data Availability

Data presented in this study are available on request from the corresponding author.
